# A new nano-encapsulated TSPO ligand reduces neuroinflammation and improves cognitive functions in Alzheimer's disease model

**DOI:** 10.7150/thno.106083

**Published:** 2025-03-21

**Authors:** Antonella Casamassa, Paola Brancaccio, Virginia Campani, Angela Corvino, Serenella Anzilotti, Giovanni Pecoraro, Giorgia Andreozzi, Raffaella Daniele, Ilaria Piccialli, Anna Pannaccione, Pierluigi Reveglia, Lucia Lecce, Carmela Paolillo, Ferdinando Fiorino, Giuseppe De Rosa, Giuseppe Caliendo, Lucio Annunziato, Giuseppe Pignataro

**Affiliations:** 1 IRCCS SYNLAB SDN, Via G. Ferraris 144, 80146 Naples, Italy; 2 Division of Pharmacology, Department of Neuroscience, School of Medicine, University of Naples Federico II, 80131, Naples, Italy; 3 Department of Pharmacy, Federico II University of Naples, Naples, Italy; 4 Department of Science and Technology University of Sannio, Benevento Italy; 5 Dipartimento di Medicina Clinica e Sperimentale, University of Foggia, Foggia, Italy

**Keywords:** TSPO, neuroinflammation, Alzheimer's disease, nanovectors, neurodegeneration

## Abstract

**Rationale:** The translocator protein 18 kDa (TSPO) is mainly expressed on the outer mitochondrial membrane and is implicated in inflammation, cell survival, and proliferation. TSPO expression in activated microglia is upregulated in Alzheimer's disease (AD), representing both a biomarker and therapeutic target for neuroinflammation.

**Methods:** We synthesized a new TSPO ligand, TEMNAP, a hybrid of temazepam, a compound well known for its ability to bind TSPO, and naproxen, a drug with anti-inflammatory properties that is potentially useful to mitigate neuroinflammation. TEMNAP was encapsulated in a self-assembling nanoparticle transferrin-targeted (SANP-TF-TEMNAP) for brain delivery. The effectiveness of TEMNAP in mitigating inflammatory processes and cognitive behavior was investigated in genetically modified Tg2576 mice, a model of Alzheimer's disease. Its effect on neuroinflammation has also been explored in lipopolysaccharide-activated BV2 microglial cells.

**Results:** SANP-TEMNAP significantly reduced the expression of proinflammatory markers in activated microglia, and this effect was abrogated by TSPO silencing. More importantly, TEMNAP was mass-spectrometrically detected in the hippocampus and cortex of Tg2576 mice after SANP-TF-TEMNAP intraperitoneal administration, preventing hippocampal neuroinflammation and improving cognitive function.

**Conclusions**: These results emphasize the following: (i) the role of transferrin-conjugated self-assembling nanoparticles (SANP-TF) as CNS nanovectors, and (ii) the potential therapeutic effectiveness of peripherally administered SANP-TF-TEMNAP in preventing neuroinflammation associated with cognitive decline.

## Introduction

Neuroinflammatory processes play a key role in the pathogenesis of Alzheimer's disease (AD). The identification of AD risk genes associated with innate immune functions and the increased levels of inflammatory markers found in patients with AD supports the crucial impact of neuroinflammation in the pathogenesis of AD and other neurodegenerative diseases 1. Among the innate immune cells, microglia is one of the primary players in neuroinflammation and microglial cells, once activated, may interact with amyloid-β and tau species thus influencing AD progression 1. A characteristic feature of activated microglia is increased expression of the mitochondrial 18-kDa translocator protein (TSPO). Mitochondrial TSPO is involved in cell proliferation and differentiation, cholesterol transport, steroidogenesis regulation, and nuclear gene expression regulation 2. When microglia are activated in response to brain injury, TSPO is upregulated; therefore, TSPO is a crucial indicator of neuroinflammation. Consequently, TSPO has been identified as an in vivo biomarker of neuroinflammation detectable via positron emission tomography (PET) and a potential target for the treatment of a range of neurodegenerative diseases, including Alzheimer's disease 3-5. In fact, TSPO overexpression or TSPO ligand treatment reduces pro-inflammatory cytokine release in lipopolysaccharide (LPS)-treated microglial cells, whereas TSPO knockdown increases cytokine release, such as RANTES and IL-12p40/p70 6-8. In addition, anti-inflammatory effects mediated by TSPO ligands have been observed in vivo in tauopathy mouse models 9-11. More interestingly, it has recently been demonstrated that TSPO ligands, such as XBD173, ameliorate AD pathology through TSPO-dependent modulation of neurosteroids 12-17, and these ligands seem to play a major role in ameliorating the energy burden occurring in AD. Considering these premises, we examined the effectiveness of a newly synthesized TSPO ligand, TEMNAP, in mitigating inflammatory processes associated with dementia. In particular, after a careful design study aimed at identifying the moieties responsible for the interaction with the protein counterpart, we focused our attention on this molecular hybrid, resulting from the fusion of the benzodiazepine nucleus of temazepam, a compound well known for its ability to bind TSPO, and naproxen, a drug with anti-inflammatory properties potentially useful to mitigate neuroinflammation. The effectiveness of TEMNAP in mitigating inflammatory processes was investigated in LPS-activated BV2 microglial cells, either alone or encapsulated in lipid nanovectors. Lipid nanovectors have been used to facilitate the internalization of encapsulated compounds 18-20. In addition, transferrin (TF) was included on the nanoparticle surface to further facilitate blood-brain barrier (BBB) crossing^21^. In fact, the binding of TF to its receptor, which is strongly expressed at the BBB level 22, improves nanoparticle central nervous system (CNS) biodistribution 23. On the other hand, self-assembling nanoparticles (SANPs) were successfully used by our group for the delivery of active compounds to the CNS 18. Compared to other lipid-based nanovectors, SANPs offer the possibility to be prepared immediately before their use, thus avoiding stability issues and facilitating the scale-up process 15, 18. Finally, the self-assembly process makes these nanovectors versatile for the encapsulation of different active substances, independent of their chemical characteristics 18.

Based on these hypotheses, we investigated the following:

(i) The ability of SANP-TF-TEMNAP to cross the BBB and be distributed in the brain regions involved in Alzheimer's pathophysiology.

(ii) Its efficacy, when peripherally administered by intraperitoneal route, to reduce neuroinflammation biomarkers, thus improving cognitive function in Tg2576 transgenic mice, an animal model mimicking Alzheimer's disease.

## Results

### Chemical synthesis of TEMNAP

**Figure 1:**
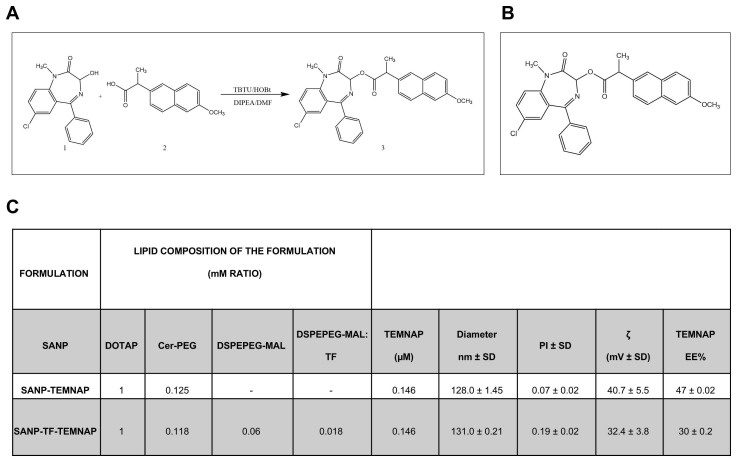
** Synthesis of molecular hybrid between Temazepam and Naproxen (TEMNAP) and physical-chemical characterization of SANPs encapsulating TEMNAP. (A-B)** Schematic representation of the chemical compound TEMNAP synthesis (3A-B) obtained by adding to the reaction mixture Temazepam (1) and Naproxen (2); **(C)** Physical-chemical characterization of SANPs encapsulating TEMNAP: the size, the polydispersity index and the zeta potential (ζ) were assessed by Zetasizer Nano Ultra (Malvern, UK).

In the present research we report the synthesis of 7-chloro-1-methyl-2-oxo-5-phenyl-2,3-dihydro-1H-benzo(e)(1,4)diazepin-3-yl2-(6-methoxynaphthalen-2-yl) propanoate (TEMNAP). The general strategy for the synthesis of TEMNAP is summarized in Figure 1A-B. Treatment of commercially available naproxen (2), with TBTU, HOBT, in presence of DMF and the subsequent addition of temazepam (1) and DIPEA stirring at room temperature, gave the corresponding TEMNAP (3) (Figure 1A-B). The synthesized final compound was analysed by ^1^H NMR, ^13^C NMR and mass spectroscopy (MS) showing that the obtained structure was consistent with the designed compound.

### Design and characterization of self-assembling lipid nanoparticle (SANP) and transferrin-conjugated self-assembling lipid nanoparticle (TF-SANP) encapsulating TEMNAP (SANP-TF-TEMNAP)

SANP and TF-targeted SANP encapsulating TEMNAP were characterized in terms of lipid nanoparticle size, zeta potential and amount of encapsulated TEMNAP. Formulation composition and characteristics are reported in Figure 1C.

SANP formulations showed a very similar size, with lipid nanoparticles mean diameter ranging from approximately 128 to 131 nm. This suggests that the presence of TF on lipid nanoparticle surface did not significantly impact on the formulation's characteristics. The nanoparticle zeta potential, whose positivity is an index of nanoparticle stability and resistance to aggregation, was positive in all cases, although the inclusion of transferrin on nanoparticle surface resulted in a slight decrease of the zeta potential, as shown in Figure 1C. Finally, TEMNAP encapsulation efficiency ranged from 30 to 47%.

### TEMNAP did not interfere with BV2 microglial cell viability and reduced in a concentration-dependent manner the pro-inflammatory activation state of LPS-stimulated BV2 microglial cells

**Figure 2:**
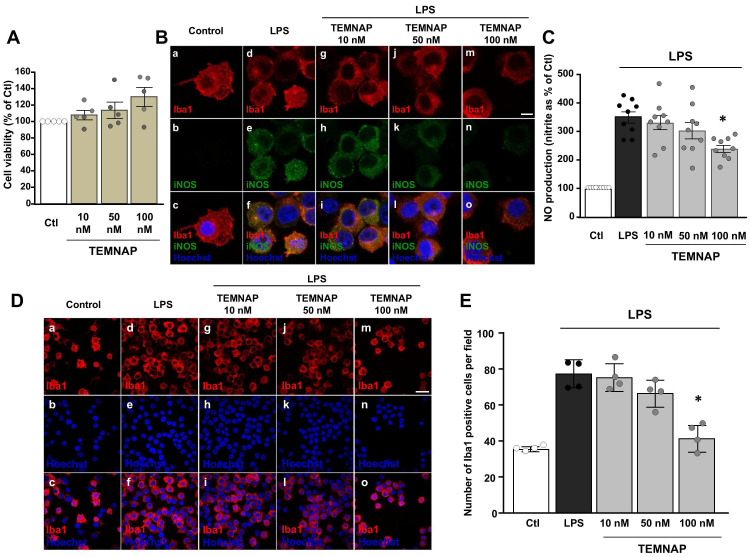
** Characterization of the new synthesized TSPO ligand TEMNAP: *in vitro* experiments to assess the concentration-response by evaluating iNOS expression, NO release and microglial cell proliferation. (A)** Effect of the new synthesized compound TEMNAP on BV2 microglia viability. Data are expressed as the percentage of control (Ctl) condition. The values represent the mean ± SEM; (n = 4 independent experimental replicates); **(B)** Representative confocal images displaying the expression of Iba1 (red) and iNOS (green) in BV2 microglia cell cultures in control condition (a-c) and in LPS-stimulated microglia in absence (d-f) or in presence (g-o) of the new synthesized compound TEMNAP (10 nM) (g-i), TEMNAP (50 nM) (j-l) and TEMNAP (100 nM) (m-o). Nuclei were stained by the nuclear dye Hoechst-33258. Scale bars: 10 µm. **(C)** NO production in BV2 microglia cell cultures in control condition and in LPS-stimulated microglia in absence or in presence of the new synthesized compound TEMNAP (10 nM), TEMNAP (50 nM) and TEMNAP (100 nM). Data are expressed as percentage of control (Ctl) condition. The values represent the mean ± SEM; (n = 9 independent experimental replicates); *p < 0.05 *versus* control. **(D)** Representative confocal images displaying the expression of Iba1 (red) in BV2 microglia cell cultures in control condition (a-c) and in LPS-stimulated microglia in absence (d-f) or in presence (g-o) of the new synthesized compound TEMNAP (10 nM) (g-i), TEMNAP (50 nM) (j-l) and TEMNAP (100 nM) (m-o). Nuclei were stained by the nuclear dye Hoechst-33258. Scale bars: 50 µm. **(E)** Quantitative analysis of Iba1-positive cells in BV2 microglia cell cultures in control (Ctl) condition and in LPS-stimulated microglia in absence or in presence of the new synthesized compound TEMNAP (10 nM), TEMNAP (50 nM) and TEMNAP (100 nM). The values represent the mean±SEM; (n = 4 independent experimental replicates); *p < 0.05 *versus* control.

The newly synthesized compound TEMNAP did not affect BV2 cell viability at the concentration of 10 nM, 50 nM or 100 nM for 24 h (Figure 2A).

Interestingly, in LPS-activated BV2 microglial cells, TEMNAP (100 nM) reduced the expression of the pro-inflammatory marker iNOS (Figure 2B) and it was able to exert an anti-proliferative effect (Figure 2D-E). More interestingly, LPS-induced production of NO was significantly reduced in BV2 microglial cells exposed to (100 nM) TEMNAP (Figure 2C).

### SANP encapsulating TEMNAP prevented microglia proliferation and reduced the expression of the pro-inflammatory markers iNOS and CD86 in LPS-stimulated BV2 microglial cells

**Figure 3:**
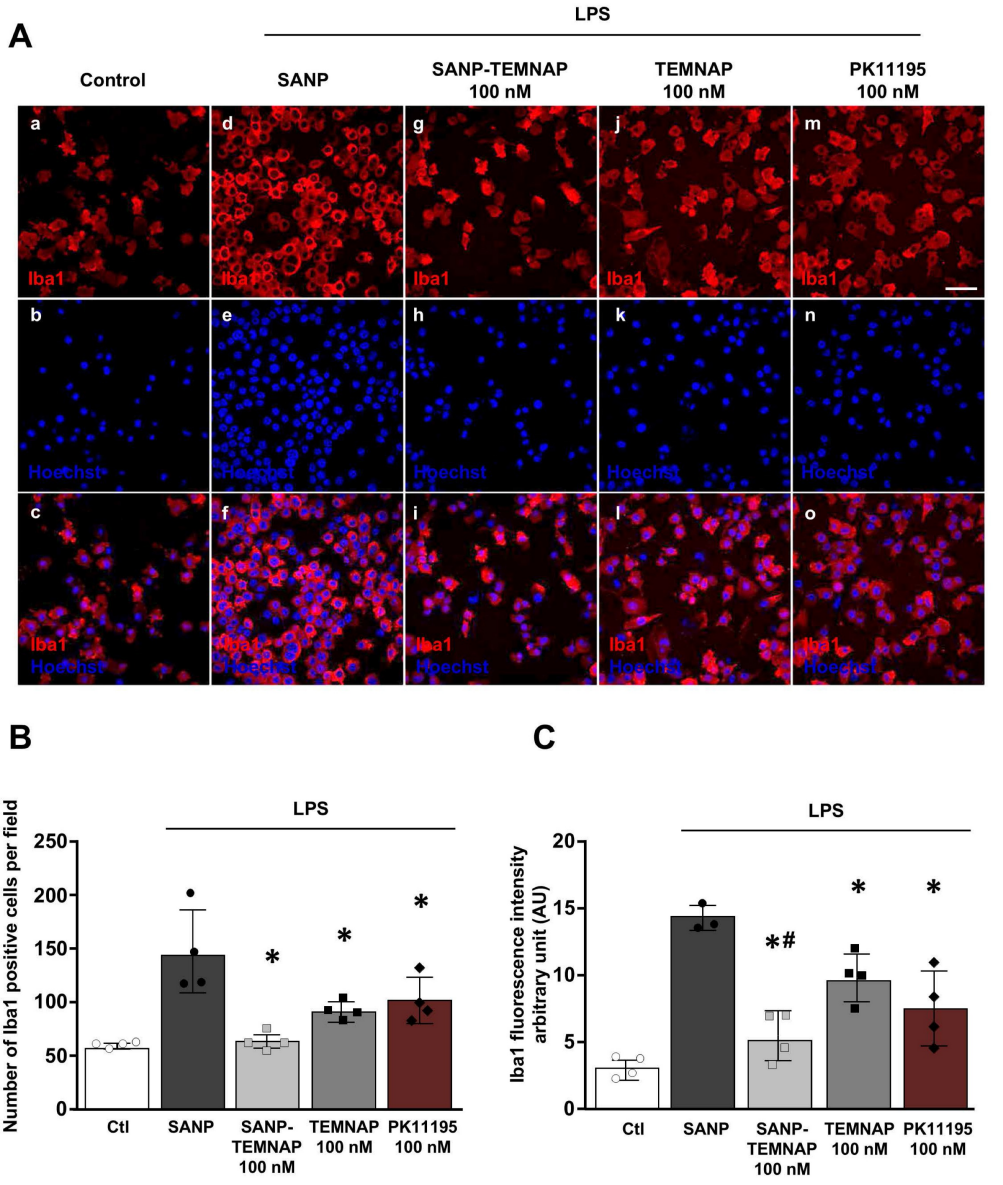
** Effect of TEMNAP (100 nM) on microglial cell proliferation and Iba1 expression after exposure of BV2 cells to LPS. (A)** Representative confocal images displaying the expression of Iba1 (red) in control condition (a-c) and in LPS-stimulated microglia (d-o) co-exposed to the following conditions: (1) self-assembling nanoparticle (SANP) (d-f); (2) nanoencapsulated-TEMNAP (100 nM) (SANP-TEMNAP) (g-i); (iii) TEMNAP (100 nM) non-nanoencapsulated (j-l); (3) the classical TSPO ligand PK11195 (m-o). Nuclei were stained by the nuclear dye Hoechst-33258. Scale bars: 50 µm. **(B)** Quantitative analysis of Iba1^)^-positive cells. The values represent the mean ± SEM; (n = 4 independent experimental replicates); *p < 0.05 *versus* LPS. **(C)** Quantitative analysis of Iba1 fluorescence intensity. The values represent the mean ± SEM; (n = 4 independent experimental replicates); *p < 0.05 *versus* control; #<0.05 versus TEMNAP (100 nM).

Quantitative immunocytochemical studies showed that TEMNAP and PK11195, a well-known TSPO ligand, exerted an anti-proliferative effects in Iba1-positive microglial cells activated by LPS (Figure 3A-B).

Interestingly, quantitative immunofluorescence studies revealed that when TEMNAP was encapsulated in nanoparticles it was able to significantly reduce LPS-induced the increase of Iba1-positive cells and the Iba1 fluorescence intensity (Figure 3B-C).

In addition, TEMNAP encapsulated in nanoparticles (SANP-TEMNAP) caused a significant reduction in the number of Iba1-positive microglial cells provided with few processes (34.92 ± 8.19%) (primed microglia) and in the number of the round-shaped hyperactivated microglial cells (0.18 ± 0.18%) (Figure S1).

**Figure 4:**
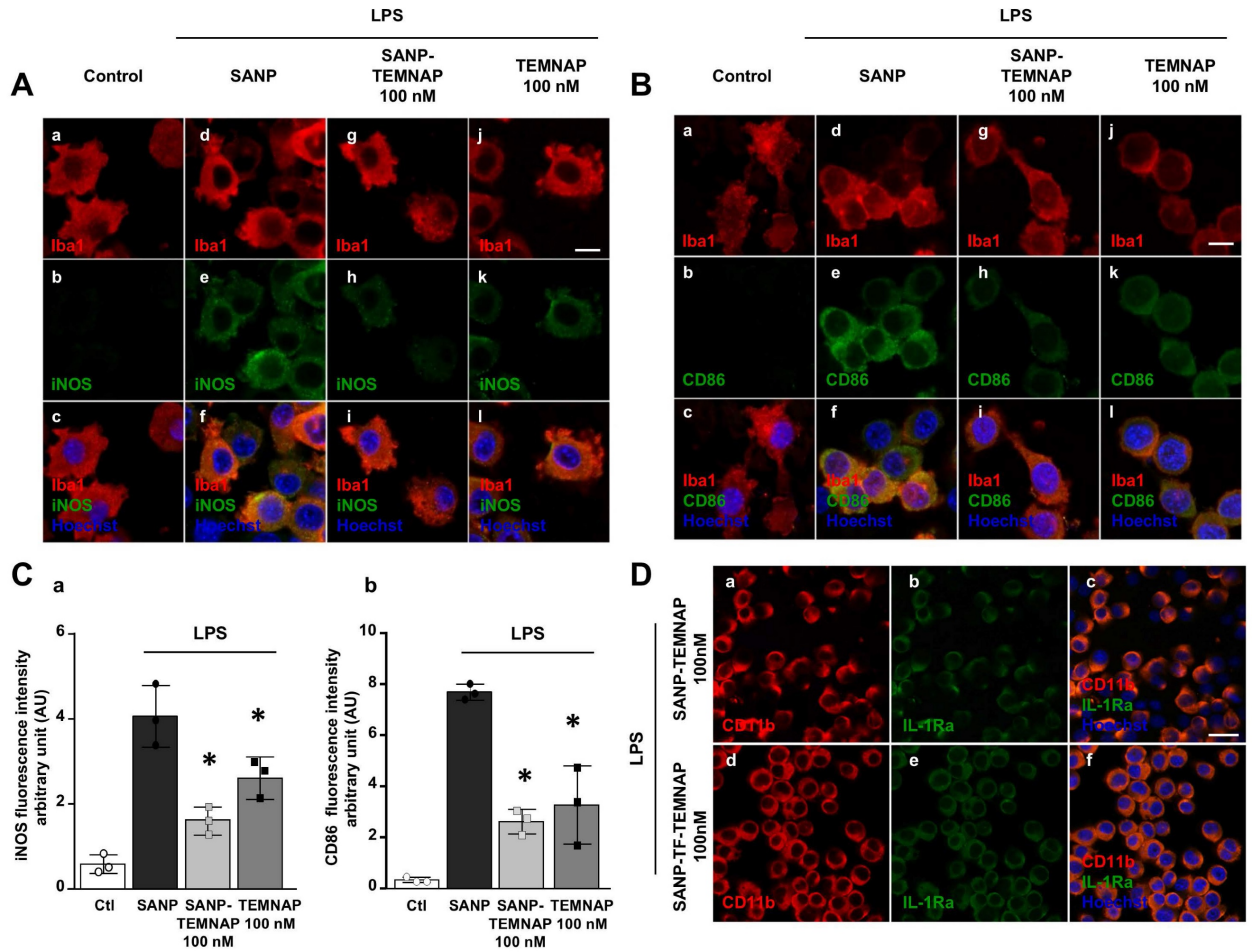
** Expression of the pro- and anti-inflammatory markers (iNOS, CD86 and IL-1Ra) in LPS-stimulated BV2 microglia exposed to SANP-TEMNAP (100 nM). (A)** Representative confocal images displaying the expression of Iba1 (red) and iNOS (green) in BV2 microglia cell cultures in control condition (a-c) and in LPS-stimulated microglia co-exposed to the following conditions: (i) self-assembling nanoparticle (SANP) (d-f); (ii) nanoencapsulated-TEMNAP (100 nM) (SANP-TEMNAP) (g-i); (iii) TEMNAP (100 nM) non-nanoencapsulated (j-l). Nuclei were stained by the nuclear dye Hoechst-33258. Scale bars: 10 µm. **(B)** Representative confocal images displaying the expression of Iba1 (red) and CD86 (green) in BV2 microglia cell cultures in control condition (a-c) and in LPS-stimulated microglia co-exposed to the following conditions: (i) self-assembling nanoparticle (SANP) (d-f); (ii) nanoencapsulated-TEMNAP (100 nM) (SANP-TEMNAP) (g-i); (iii) TEMNAP (100 nM) non-nanoencapsulated (j-l). Nuclei were stained by the nuclear dye Hoechst-33258. Scale bars: 10 µm. **(C)** Quantitative analysis of iNOS (a) and CD86 (b) fluorescence intensities. The values represent the mean±SEM; (n = 3 independent experimental replicates); *p < 0.05 *versus* control. **(D)** Representative confocal images displaying the expression of CD11b (red) and IL-1Ra (green) in BV2 microglia cell cultures in LPS-stimulated microglia co-exposed to the following conditions: (i) nanoencapsulated-TEMNAP (100 nM) (SANP-TEMNAP) (a-c); (ii) transferrin-conjugated nanoparticles containing TEMNAP (100 nM) (SANP-TF-TEMNAP) (d-f); Nuclei were stained by the nuclear dye Hoechst-33258. Scale bars: 20 µm.

Interestingly, 100 nM TEMNAP, either in free form or delivered by nanoparticles (SANP), significantly reduced the expression of the pro-inflammatory markers iNOS (Figure 4A-C) and CD86 (Figure 4B-C) in LPS-stimulated microglial cells. More interestingly, transferrin-conjugated nanoparticles containing TEMNAP (SANP-TF-TEMNAP) slightly promoted the expression of the microglial anti-inflammatory marker IL-1Ra (Figure 4D) if compared to LPS-stimulated microglia exposed to non-conjugated nanoparticles containing TEMNAP (SANP-TEMNAP). These results showed that the nanoparticle strategy did not interfere with, but rather reinforced, the pharmacological effect of the newly synthesized compound TEMNAP.

### TSPO silencing prevented the decrease in iNOS expression and reduction of pro-inflammatory cytokine release induced by SANP-TEMNAP in LPS-stimulated microglial cells

**Figure 5:**
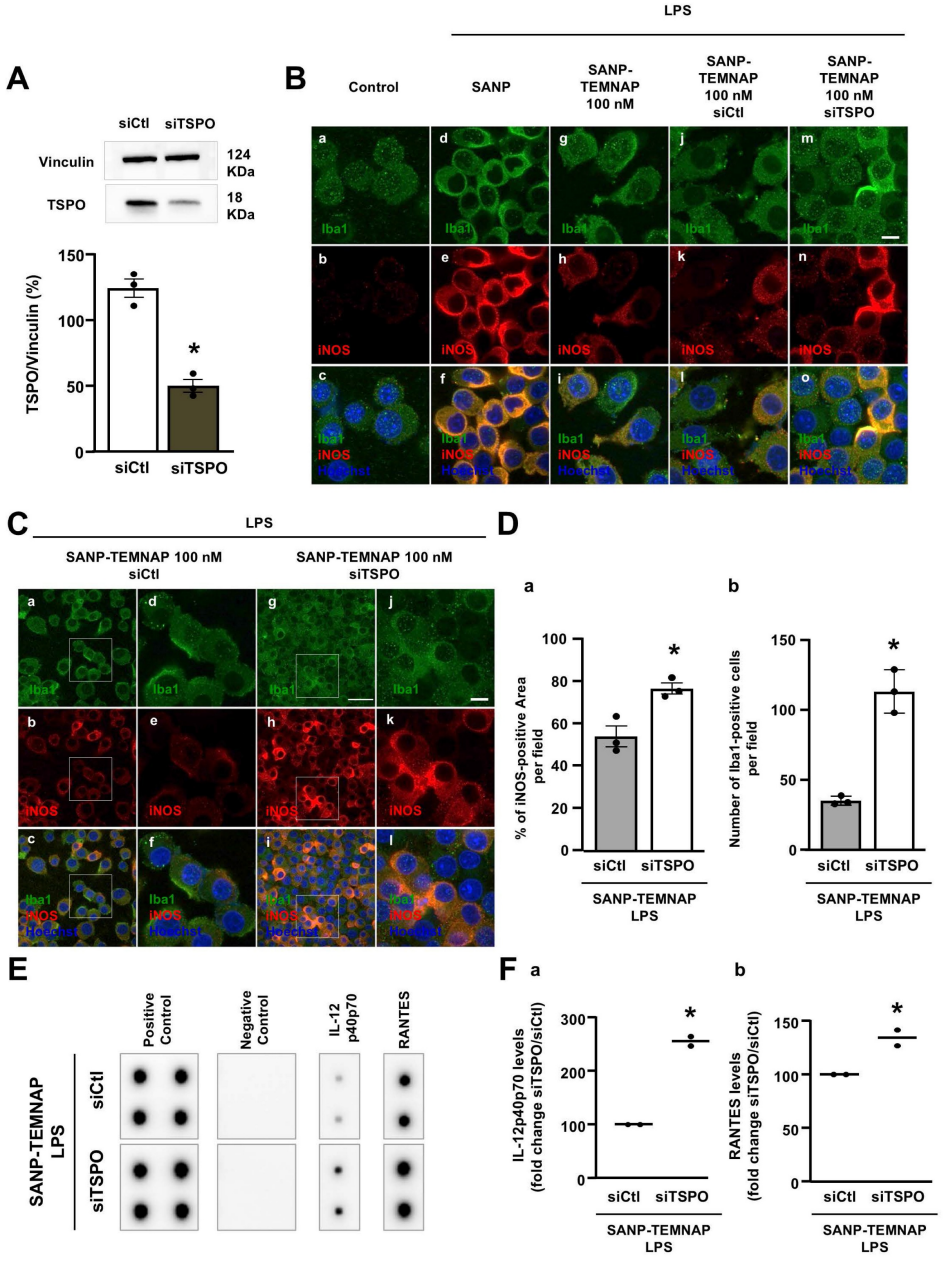
** Evaluation of LPS-induced proliferation, iNOS expression and cytokine release in BV2 microglial cells co-treated with the nanoparticle-encapsulated TEMNAP (100 nM) in presence of siTSPO (100 nM) or in presence of siControl (100 nM) for 72 h. (A)** Representative blotting and quantitative densitometric analysis of TSPO protein expression levels in BV2 microglial cells exposed to siRNA targeting tspo gene (siTSPO) (100 nM) or to siControl (siCtl) for 72 h. The values represent the mean±SEM; (n = 3 independent experimental replicates); *p < 0.05 *versus* siControl. **(B)** Representative confocal images displaying the expression of Iba1 (green) and iNOS (red) in BV2 microglia cell cultures in control condition (a-c) and in LPS-stimulated microglia exposed to self-assembling nanoparticle (SANP) (d-f); nanoencapsulated-TEMNAP (100 nM) (SANP-TEMNAP); nanoencapsulated-TEMNAP (100 nM) in presence of siControl (100 nM) (j-l); nanoencapsulated-TEMNAP (100 nM) in presence of siTSPO (100 nM). Nuclei were stained by the nuclear dye Hoechst-33258. Scale bars: 10 µm. **(C)** Representative confocal images displaying the expression of Iba1 (green) and iNOS (red) in BV2 microglia cell cultures in LPS-stimulated microglia co-exposed to nanoencapsulated-TEMNAP in presence of siCtl (a-f); nanoencapsulated-TEMNAP (100 nM) in presence of siTSPO (g-l). Panels d,e,f represent higher magnification of the panels a,b and c respectively. Panels j,k,l represent higher magnification of the panels g,h and i respectively. Nuclei were stained by the nuclear dye Hoechst-33258. Scale bars: 10 µm. **(D)** Quantification of iNOS-fluorescence positive area (a) and quantitative analysis of Iba1-positive cells (b) in LPS-stimulated BV2 microglia co-exposed to nanoencapsulated-TEMNAP (100 nM) in presence of siControl (100 nM) and nanoencapsulated-TEMNAP (100 nM) in presence of siTSPO (100 nM). The values represent the mean ± SEM; (n = 3 experimental replicates); *p < 0.05 *versus* siControl. **(E)** Cytokine antibody array membrane representing the expression levels of two pro-inflammatory cytokines, IL-12p40p70 and RANTES, in the culture media of LPS-stimulated BV2 microglial cells co-exposed to SANP-TEMNAP in presence of siCtl and siTSPO. **(F)** Quantitative densitometric analysis representing the expression levels of IL-12p40p70 (a) and RANTES (b), in the culture media of LPS-stimulated BV2 microglial cells co-exposed to SANP-TEMNAP in presence of siCtl and siTSPO. Data are expressed as the percentage of siControl. The values represent the mean of two spots for each cytokine; *p < 0.05 *versus* siControl.

The involvement of the TSPO protein as a specific target of TEMNAP was confirmed through TSPO silencing using a selective siRNA (Figure 5A; Figure S2). Double immunocytochemical experiments conducted with the microglial marker Iba1 in the presence of the pro-inflammatory marker iNOS demonstrated that TSPO silencing prevented the reduction of iNOS immunosignal observed in LPS-stimulated microglia exposed to nanoparticles encapsulating TEMNAP (Figure 5B; Figure 5D). Furthermore, quantitative studies revealed that TSPO silencing completely prevented the anti-proliferative effect observed in LPS-stimulated microglia following treatment with SANP-encapsulating TEMNAP (Figure 3B; Figure 5C-D). The endogenous microglial TSPO protein, as a target of the anti-inflammatory compound TEMNAP, was further confirmed through pro-inflammatory cytokine expression experiments utilizing a cytokine antibody array. Notably, when TSPO was silenced in LPS-stimulated BV2 microglial cells, the reduction in the pro-inflammatory cytokines RANTES and IL-12p40/p70, induced by the encapsulated compound TEMNAP, was inhibited (Figure 5E-F).

### TEMNAP was detected by mass spectrometry in the hippocampus and cortex of Tg2576 mice, peripherally administered by the intraperitoneal route with SANP-TF-TEMNAP

To verify the ability of SANP-TF-TEMNAP to cross the BBB and reach the brain regions of interest, the TEMNAP content was measured by mass spectrometry after intraperitoneal administration. The amount of TEMNAP was 1.113 ± 0.115 mg/g of tissue in the cortex and 0.015 ± 0.0018 mg/g of tissue in the hippocampus. No detection was observed in the cortex or hippocampus of animals treated with empty or non-encapsulated TEMNAP. An example of the chromatogram and mass spectrum is shown in Figure S3.

### Intraperitoneally infused transferrin-conjugated nanoparticles containing TEMNAP (SANP-TF-TEMNAP) reduced the expression of the pro-inflammatory markers CD86 and iNOS in the hyperactivated microglia of the hippocampus of Tg2576 mice

**Figure 6:**
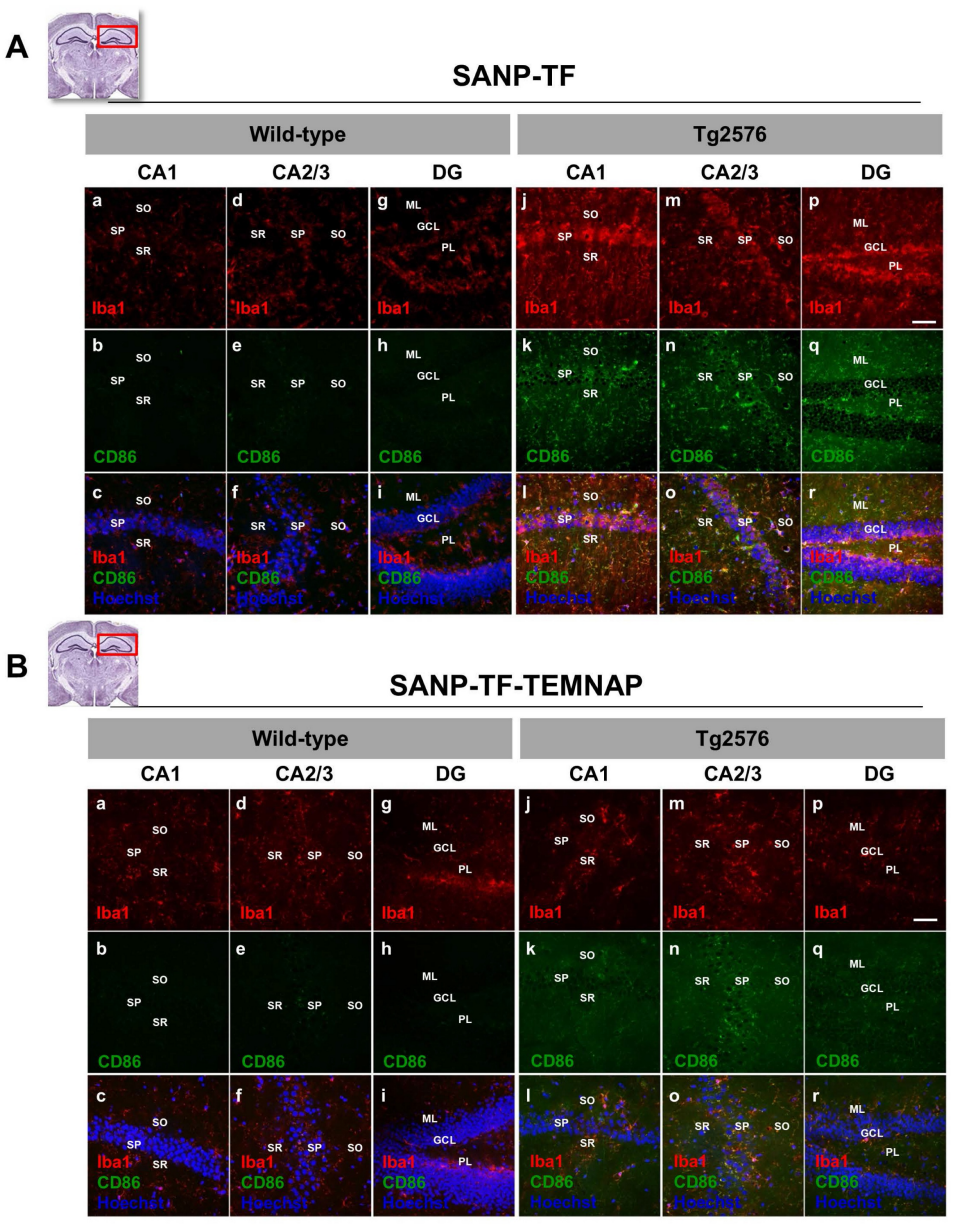
** Evaluation of Iba1 and CD86 expression in hippocampus of Tg2576 mice chronically treated with TEMNAP encapsulated in a transferrin-engineering precision nanoparticle system (SANP-TF-TEMNAP).** Representative brain slice cartoons indicating the area of interest are on the top side of the Figures.** (A)** Confocal images displaying the expression of the marker Iba1 (red) and the pro-inflammatory marker CD86 (green) in the hippocampal regions of wild-type (a-i) and Tg2576 (j-r) mice treated with the empty transferrin-engineering nanoparticles (SANP-TF) or **(B)** treated with transferrin-engineering nanoparticles containing TEMNAP (SANP-TF-TEMNAP). Sections were captured in the different hippocampal regions: CA1, CA2, CA3 and Dentate Gyrus (DG) -. Nuclei were stained by the nuclear dye Hoechst-33258. Stratum Oriens (SO), Stratum Pyramidale (SP), Stratum Radiatum (SR), Molecular Layer (ML), Granular Cell Layer (GCL), Polymorphic Layer (PL) Scale bars: 50 µm.

**Figure 7:**
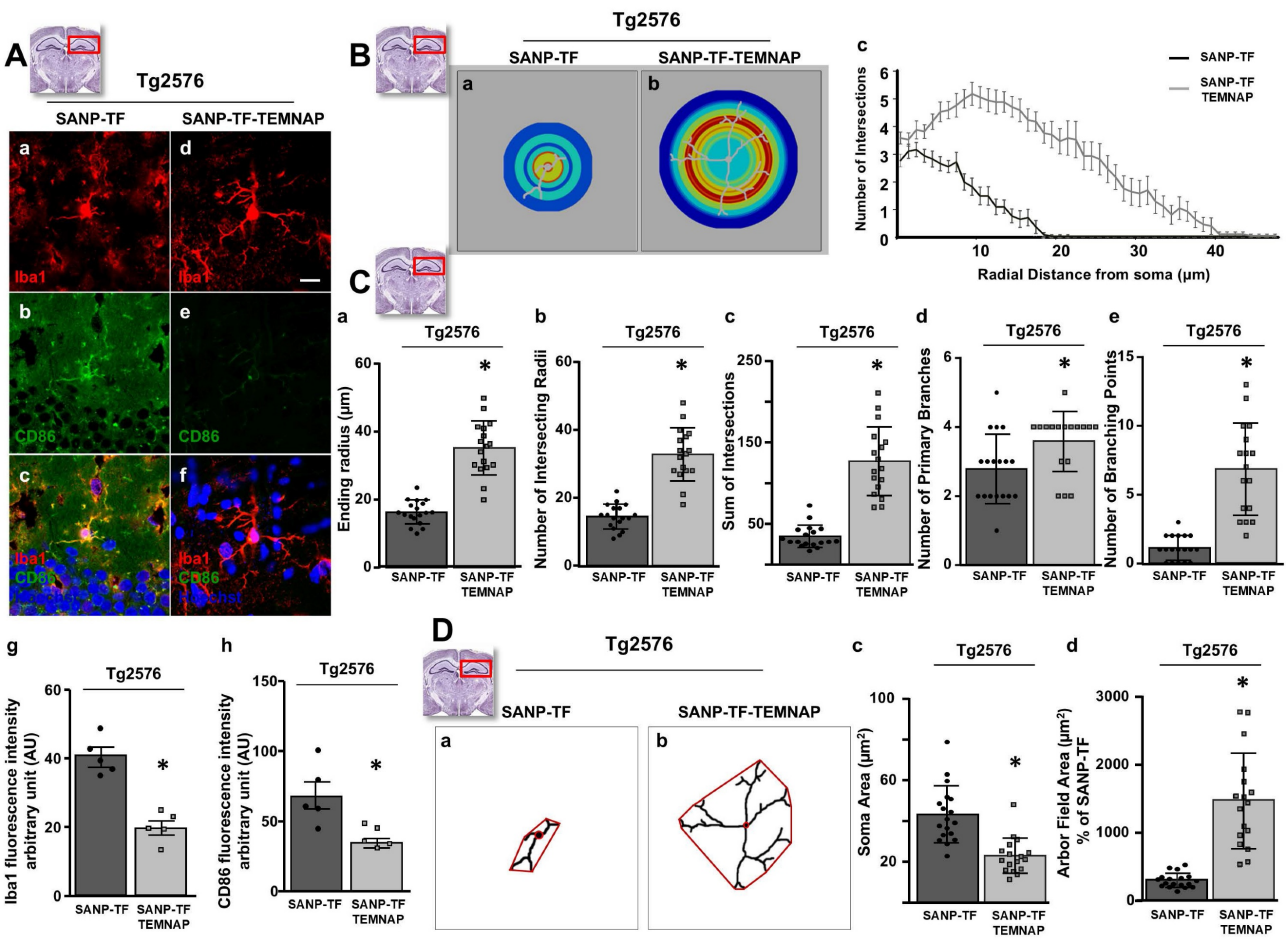
** Morphological characterization of Iba1-positive microglia in the hippocampus of Tg2576 mice chronically treated with SANP-TF or SANP-TF-TEMNAP. (A)** Representative confocal images displaying the hippocampal microglial expression of Iba1 (a,d), CD86 (b,e) and Merge (c,f) from Tg2576 mice treated with SANP-TF or with SANP-TF-TEMNAP. Nuclei were stained by the nuclear dye Hoechst-33258. Scale bars: 20 µm. Quantitative analysis of Iba1 (g) and CD86 (h) fluorescence intensity in brain sections from Tg2576 mice treated with SANP-TF or with SANP-TF-TEMNAP. The values represent the mean ± SEM; (n = 5 independent experiments); *p < 0.05 *versus* control. **(B)** (a-b) Representative pictures of Sholl masks of individual Iba1-positive hippocampal microglial cells. (SANP-TF, n = 18 from 3 independent experiments; SANP-TF-TEMNAP, n = 18 from 3 independent experiments). Scale bars: 20 µm. (c) Single-cell microglia morphology reconstruction by Sholl analysis. Data are represented in a line graph as mean ± SEM (SANP-TF, n = 18 from 3 independent experiments; SANP-TF-TEMNAP, n = 17 from 3 independent experiments). **(C)** Quantitative evaluation of hippocampal microglial morphology: (a) ending radius, (b) intersecting radii, (c) sum of intersections, (d) primary branches, (e) branching point. Data are expressed as mean ± SEM (SANP-TF, n = 18 from 3 independent experiments; SANP-TF-TEMNAP, n = 17 from 3 independent experiments). **(D)** (a-b) Representative examples showing the binary images of microglial morphology reconstructed starting from Iba1 immunoreactivity. In red are respectively indicated the soma cells and microglial arbor field area. Scale bars: 20 µm. (c-d) Quantitative evaluation of cell morphology performed by analyzing: (c) the soma area (in µm^2^) and (d) the microglial arbor field area (in µm^2^), Data are expressed as mean ± SEM (SANP-TF, n = 18 from 3 independent experiments; SANP-TF-TEMNAP, n = 17 from 3 independent experiments). Representative brain slices on the top of the figure represent the hippocampal area of interest.

**Figure 8:**
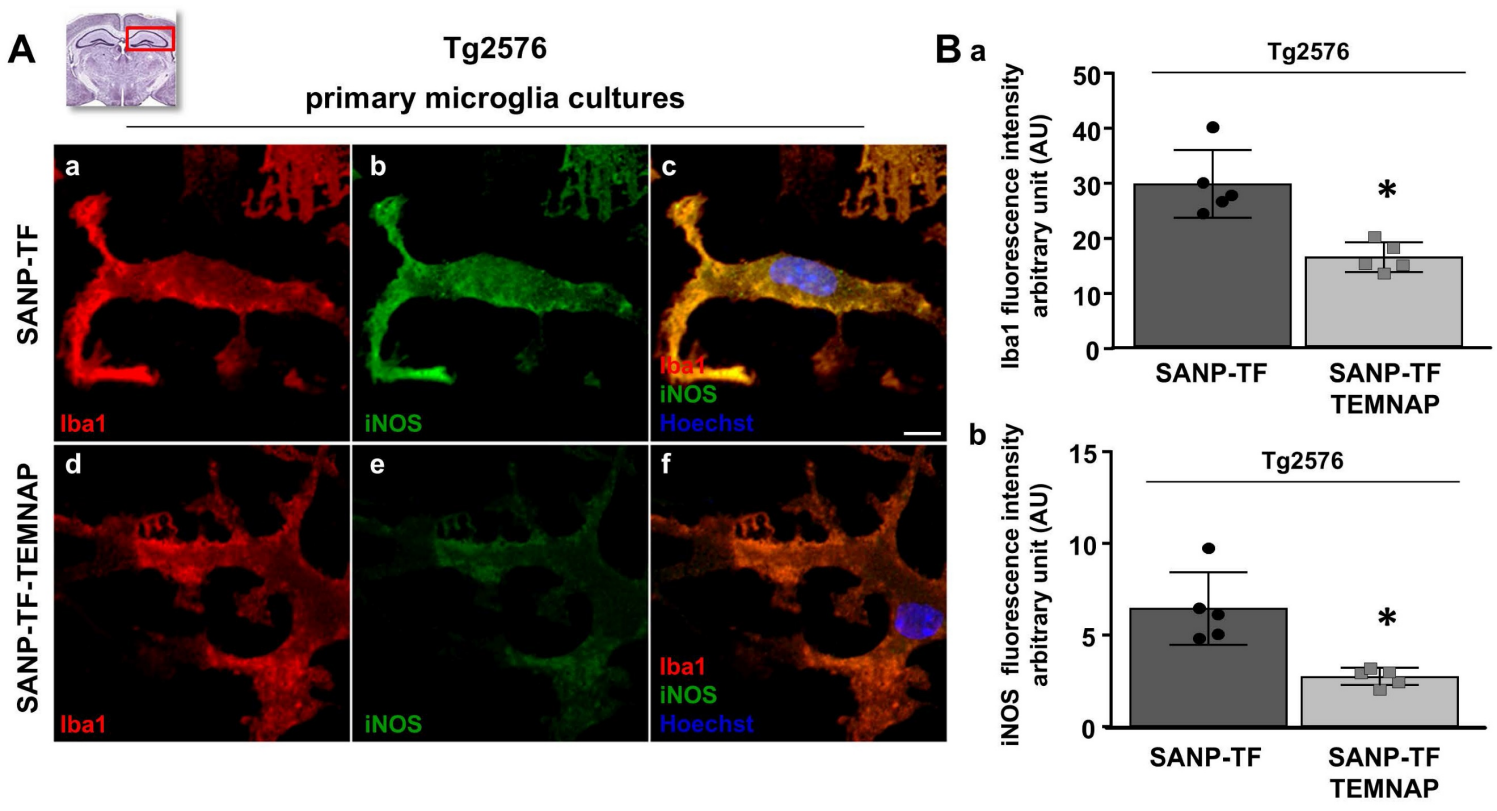
** Evaluation of Iba1 and iNOS expression in primary *ex vivo* microglia cultures obtained from hippocampus of Tg2576 mice chronically treated with with SANP-TF or SANP-TF-TEMNAP. (A)** Representative confocal images displaying the microglial expression of Iba1 (a,d) and iNOS (b,e) and merge (c,f) in *ex vivo* primary cultures from Tg2576 mice chronically treated with SANP-TF or with SANP-TF-TEMNAP. Nuclei were stained by the nuclear dye Hoechst-33258. Scale bars: 10 µm. **(B)** Quantitative analysis of Iba1 (a) and iNOS (b) fluorescence intensity in *ex vivo* primary cultures from Tg2576 mice chronically treated with SANP-TF or with SANP-TF-TEMNAP. The values represent the mean±SEM; (n = 5 indipendent experiments); *p < 0.05 versus control. A representative brain slice on the top of the figure represents the hippocampal area of interest.

To confirm the in vitro results obtained in BV2 microglia, we performed immunohistochemical studies in transgenic Alzheimer's Tg2576 mice chronically and peripherally administered SANP-TF-TEMNAP. The presence of transferrin on nanoparticles was adopted to enhance TEMNAP crossing of the BBB, which strongly expresses transferrin receptors (TfR). Interestingly, quantitative immunohistochemical experiments showed that the expression of CD86 was significantly reduced in Iba1-positive cells in the dentate gyrus, CA1, and CA2/3 of the hippocampus of transgenic mice Tg2576 peripherally treated with transferrin-conjugated nanoparticles containing TEMNAP (Figure 6). More interestingly, in the same hippocampal regions, there was a strong reduction in the activation of microglial cells, as evaluated by quantitative morphometric analyses of cell arborization complexity (Figure 7). The pro-inflammatory microglial state was also investigated in *ex vivo* cultured microglial cells obtained from the hippocampi of Tg2576 mice chronically and peripherally treated with SANP-TF-TEMNAP. In these *ex vivo* cells, a significant reduction in iNOS fluorescence intensity was detected in Iba1-positive cells (Figure 8), confirming the anti-neuroinflammatory effect of TEMNAP.

### Chronic peripheral SANP-TF-TEMNAP administration ameliorated spatial and recognition memory in transgenic Tg2576 mice

To investigate the effect of SANP-TF-TEMNAP on spatial and recognition memory, a series of cognitive tests were performed, including the T-maze spontaneous alternation, object recognition, and Barnes maze tests.

**Figure 9:**
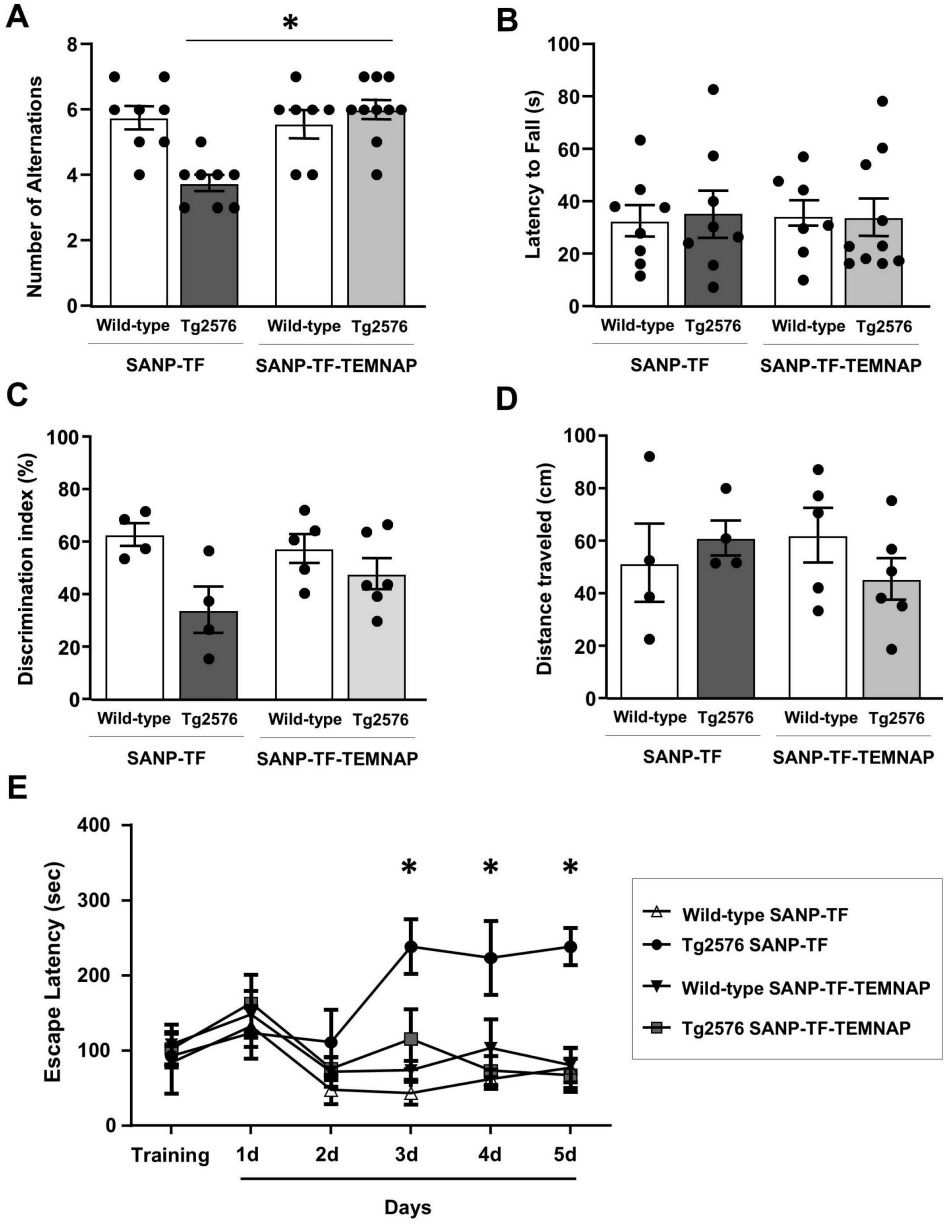
** Evaluation of cognitive impairment in Tg2576 mice chronically treated with SANP-TF or SANP-TF-TEMNAP. (A)** Total number of alternations from T-Maze test in the Tg2576 mice chronically treated with SANP-TF or with SANP-TF-TEMNAP. **(B)** Latency to fall from T-Maze test, the data are expressed in s. The values represent the mean±SEM; (n = 8/10 for each group); *p < 0.05 *versus* SANP-TF-TEMNAP treated Tg2576 mice. **(C)** Discrimination index from object recognition test in the Tg2576 mice chronically treated with SANP-TF or with SANP-TF-TEMNAP. The data are expressed in percentage. **(D)** Distance traveled in the object recognition test are expressed in cm. The values represent the mean±SEM; (n = 4/6 for each group); *p < 0.05 *versus* SANP-TF treated wild-type mice. **(E)** Latency to escape from Barnes maze test in the Tg2576 mice chronically treated with SANP-TF or with SANP-TF-TEMNAP. The data are expressed in s. The values represent the mean±SEM; (n = 4/6 for wild-type SANP-TF and wild-type SANP-TF-TEMNAP and n = 5/8 for Tg2576 SANP-TF and Tg2576 SANP-TF-TEMNAP); *p < 0.05 *versus* all experimental groups.

Repetitive behavior and spatial working memory were evaluated using a T-maze test. As expected, Tg2576 mice treated with empty SANP-TF showed a lower number of alternations between the two arms of the T-maze than wild-type mice. In contrast, when Tg2576 mice were chronically administered SANP-TF-TEMNAP, the number of alternations increased by 20% and returned to values comparable to those obtained in healthy wild-type animals (Figure 9A). In contrast, the latency time, expressing the interval to enter the chosen arm, was not influenced by any of the treatments in any of the four experimental groups (Figure 9B).

To evaluate recognition memory and response to a novel object, mice were assayed in an open field cage with two objects for exploration. The difference in the amount of time spent exploring the novel object rather than the familiar one (the discrimination index) is indicative of long-term memory. As expected, the discrimination index significantly decreased in Tg2576 mice treated with empty SANP-TF without TEMNAP compared to that in SANP-TF-treated wild-type mice (Figure 9C-D) thus indicating reduced memory. Interestingly, the discrimination index was increased in Tg2576 mice treated with SANP-TF-TEMNAP. In particular, four out of six mice reached the behavioral scores observed in healthy wild-type animals (Figure 9C).

Notably, the distance traveled in the open field did not change among all the experimental groups, indicating that pharmacological treatment did not interfere with motor performance (Figure 9D).

Finally, learning and short/long memory performances were evaluated using the Barnes circular maze test, a hippocampus-dependent cognitive task that requires spatial reference memory. Tg2576 mice treated with empty SANP-TF did not learn to locate the escape hole during the observation period (5 days), suggesting an impairment of spatial learning and long-term memory performance (Figure 9E). In contrast, the escape latency observed in SANP-TF-TEMNAP-treated Tg2576 mice was comparable to that in wild-type mice (Figure 9E) thus demonstrating the efficacy of peripherally administered SANP-TEMNAP treatment on learning and short/long memory.

## Discussion and Conclusions

In the present study, combining *in vitro*, *in vivo*, and *ex vivo* experimental approaches, we developed a transferrin-conjugated self-assembling nanoparticles (SANP-TF) functioning as a CNS cargo for the new TSPO ligand, TEMNAP, which is able to reduce neuroinflammatory processes and ameliorate cognitive behavior in genetically modified Tg2576 mice, an animal model mimicking Alzheimer's disease.

Here, we demonstrated that: (i) transferrin-conjugated self-assembling nanoparticles (SANP-TF) may function as CNS cargo; (ii) the newly synthesized compound TEMNAP, which has a good cellular toxicity profile, significantly reduced the pro-inflammatory activation state of LPS-stimulated microglial cells, and this effect was abrogated by TSPO silencing; and (iii) TEMNAP was mass-spectrometrically detected in the hippocampus and cortex of Tg2576 mice after SANP-TF-TEMNAP intraperitoneal administration and reduced neuroinflammation, thus ameliorating cognitive decline in genetically modified Tg2576 mice. Neuroinflammation was also significantly reduced in *ex vivo* primary microglial cultures obtained from SANP-TF-TEMNAP treated Tg2576 mice.

Our results strongly support previous findings in which TSPO ligands attenuated microgliosis and neuroinflammation both *in vitro* and *in vivo* in neurological disorders, including Alzheimer's disease (9-17, 35-36) thus confirming that TSPO represents a promising target for the development of biomarkers and therapeutic strategies for neuroinflammation and cognitive decline. TSPO ligands have recently been widely used as *in vivo* biomarkers of neuroinflammation, detectable via positron emission tomography (PET) 3-5.

With regard to the mechanism of TEMNAP in reducing neuroinflammation, we found that the transferrin-conjugated nanoparticles containing TEMNAP significantly decreased in LPS-stimulated microglia, the expression of the pro-inflammatory markers iNOS and CD86, as well as the production of pro-inflammatory mediators such as nitric oxide and the cytokines IL-12p40p70 and RANTES. More importantly, SANP-TF-TEMNAP, chronically administered to Tg2576 mice, reduced the expression of the pro-inflammatory marker CD86 in CA1, CA2/3, and in the dentate gyrus of the hippocampus, and iNOS in *ex vivo* primary hippocampal microglia obtained from Tg2576 SANP-TF-TEMNAP chronically and peripherally treated mice.

In accordance with these results, it has been previously reported that other TSPO ligands, such as PK11195 and Ro-5-4864, reduce microgliosis 35-39 and pro-inflammatory cytokine release 9-17. On the other hand, it cannot be excluded that there is a possibility that, similarly to other TSPO ligands, TEMNAP could exert these neuroprotective effects also through other mechanisms such as production of neurosteroids 40-42 and reduction of the nuclear factor-kappa B (NFKB) 6, 43, 44.

Notably, considering that TEMNAP has been designed as a hybrid of the benzodiazepine temazepam, a TSPO ligand, and the nonsteroidal anti-inflammatory drug (NSAID) naproxen, it cannot be excluded that the observed pharmacological effect can also be in part attributed to naproxen properties, as previously suggested by Zhang and colleagues in an updated meta-analysis of cohort studies 45.

The observed key role of transferrin-conjugated nanoparticles as cargo for potentiating TEMNAP efficacy confirmed the potential role of the transferrin receptor as a vector to target and cross the BBB 21-23. On the other hand, we have previously shown the ability of SANP-TFs to cross the BBB and release their content in the CNS 18, 45, 46. Interestingly, TfR levels and TfR-dependent internalization mechanisms are preserved in the presence of Aβ and tau neuropathology, supporting the potential of TfR as a vector target for drug delivery in AD 9-23. Notably, in our* in vitro* results, we demonstrated that the transferrin-conjugated nanoparticle strategy favors not only BBB crossing but also cellular internalization into microglia and TEMNAP release from the nanoparticles. Furthermore, our in vivo results demonstrated that TEMNAP was detected by mass spectrometry in the hippocampus and cortex of Tg2576 mice when peripherally administered SANP-TF-TEMNAP via the intraperitoneal route 24 h before measurement. On the other hand, the diffusion of NP from the peritoneal cavity depends on different factors, including the characteristics of the delivery system 15. In fact, self-assembling NP can be assimilated into lipid nanovectors for which effective clearance from the peritoneal cavity has already been observed 15. In the design of the delivery system, transferrin was used to enhance the crossing of blood brain barrier. In addition, it should be taken into consideration that diffusion from the peritoneal cavity to the systemic circulation of NP is strongly enhanced in the case of cationic liposomes, as shown by Pan *et al.*, 2024 15. The SANP used in our experiments contained cationic lipids.

More importantly, in our study, TEMNAP, measured by mass spectrometry, was found in the brain following intraperitoneal administration, thus demonstrating that the NPs can cross the BBB after intraperitoneal administration in adequate amounts 15.

Another aspect that should be underlined is that SANP-TF-TEMNAP was administered to Tg2576 mice at the age of 7 months, when signs of cognitive decline were already present 47-50, but microgliosis was only in the initial phase. Indeed, Tg2576 mice exhibit age-associated cognitive deficits starting at 5-6 months, whereas microglial activation is more evident at 7-9 months 47, 50. In addition, it should be emphasized that the protective effect was already evident one month after peripheral TEMNAP treatment interruption.

Morphological analysis of microglia in the hippocampal regions involved in spatial and learning memory revealed that SANP-TF-TEMNAP treatment in Tg2576 mice strongly prevented microglial cellular process retraction and preserved a high number of resting and ramified microglia.

Interestingly, the observed effects mediated by SANP-TF-TEMNAP on preventing both the expression of pro-inflammatory markers and the hyperactivation of hippocampal microglia in the CA1, CA2/3, and dentate gyrus were associated with the amelioration of spatial and long-term memory in SANP-TF-TEMNAP-treated Tg2576 mice.

Our results highlight the role of transferrin-conjugated self-assembling nanoparticles (SANP-TF) as CNS cargo and the potential therapeutic effectiveness.

## Methods

### Materials

1,2-dioleoyl-3-trimethylammonium-propane chloride (LIPOID DOTAP-CL or DOTAP), N-palmitoyl-sphingosine-1 {succinyl(methoxy(polyethylene glycol)2000)} (cer-PEG2000) (cer-PEG), and 1,2-distearoyl-sn-glycero-3-phosphoethanolamine-N-(maleimide(polyethylene glycol)-2000) (ammonium salt) were purchased from Spectra 2000 s.r.l. (Rome, Italy). Transferrin was obtained from Sigma-Aldrich Co. (Milan, Italy).

### Synthesis: General procedures

All reagents were commercial products purchased from Aldrich with only exception of Temazepam that was kindly provided by GENETIC Spa - Fisciano (SA). Melting points were determined using a Kofler hot-stage apparatus and are uncorrected. ^1^H-NMR and ^13^C-NMR spectra were recorded on Varian Mercury Plus 400 MHz instrument. Unless otherwise stated, all spectra were recorded in DMSO-d6. Chemical shifts are reported in ppm using Me4Si as internal standard. The following abbreviations are used to describe peak patterns when appropriate: s (singlet), d (doublet), t (triplet), m (multiplet), q (quartet), qt (quintet), dd (double doublet), ddd (double dd), bs (broad singlet). Mass spectra of the final products were performed on API 2000 Applied Biosystem mass spectrometer. Where analyses were indicated only by the symbols of the elements, results obtained were within ± 0.4% of the theoretical values. All reactions were followed by TLC, carried out on Merck silica gel 60 F254 plates with fluorescent indicator and the plates were visualized with UV light (254 nm). Preparative chromatographic purifications were performed using silica gel column (Kieselgel 60). Solutions were dried over Na_2_SO_4_ and concentrated with Buchi rotary evaporator at low pressure.

### Synthesis of 7-chloro-1-methyl-2-oxo-5-phenyl-2,3-dihydro-1H-benzo(e)(1,4) diazepin-3-yl2-(6-methoxynaphthalen-2-yl)propanoate (TEMNAP)

The reaction useful to synthetize the compound TEMNAP is shown in a schematic representation in the Figure 1. In a 250 mL round-bottomed flask, Naproxen (**2**) (0.766 g, 0.00332 mol), TBTU (1.281 g, 0.00400 mol, 1.2 equiv.) and HOBT (0.611 g, 0.00400 mol, 1.2 equiv.) were solubilized in DMF (20 mL). After 5 min, Temazepam (**1**) (1 g, 0.00332 mol, 1 equiv.) and DIPEA (0.860 g, 0.00665 mol, 2 equiv.) were added to the reaction mixture then stirred overnight at room temperature. Subsequently, the solvent was removed under vacuum. The crude product was purified by silica gel open chromatography using dichloromethane/methanol (9:1 v/v). The final compound (**3**) was then crystallized from hexane yielding the desired product (Figure 1A-B).

**7-chloro-1-methyl-2-oxo-5-phenyl-2,3-dihydro-1H-benzo(e)(1,4) diazepin-3-yl2-(6-methoxynaphthalen-2-yl)propanoate (3).** Yield: 43%; mp: 192-194°C; ^1^H-NMR (400 MHz, DMSO‑d6) 1.55 (s, 3H,-CH_3_); 3.35 (s, 3H, -NCH_3_); 3.87 (s, 3H,-OCH_3_); 4.17 (m, 1H, CH); 5.87 (s, 1H,-CH-); 7.17 (dd, 1H, J=8.2, 2.1 Hz); 7.27 (dd, 1H, J=13.3, 2.2 Hz); 7.43-7.59 (m, 9H); 7.71 (t, 1H); 7.81 (d, 1H, J=8.5 Hz); 7.85 (s, 1H); ^13^C-NMR (100 MHz, DMSO‑d6) δ: 13.94, 15.15, 18.72, 19.98, 22.04, 30.94, 34.84, 44.25, 55.17, 64.90, 85.26, 105.73, 118.72, 124.46, 125.87, 126.52, 126.90, 128.45, 128.82, 129.50, 131.20, 132.27, 133.35, 135.29, 136.66, 141.29, 157.20, 164.31, 164.91, 173.26; ESI-MS m/z (M+H)^+^ calculated for C_30_H_25_ClN_2_O_4_ 512.15; Found = 513.25 (ESI-MS: 513.25 (M+H)^+^; 535.27 ( +Na)^+^; 551.22 (M+K)^+^).

Anal. Calc. for C_30_H_25_ClN_2_O_4_: C, 70.24; H, 4.91; N, 5.46; Found: C, 70.45; H, 4.90; N, 5.44.

### Preparation of self-assembling nanoparticles and transferrin-conjugated nanoparticles encapsulating TEMNAP (SANP-TEMNAP and SANP-TF-TEMNAP)

Self-assembling nanoparticles (SANP) encapsulating TEMNAP were prepared according to a previously reported method with some modifications 48-49. Initially, PEGylated cationic liposomes were prepared using the lipid compositions DOTAP/cer-PEG (1:0.125 mM) and TEMNAP (0.146 mM). In the case of targeted liposomes, a lipid composition of DOTAP/cer-PEG/ DSPEPEG-MAL (1:0.118:0.06) was used. After liposomes preparation, the unencapsulated TEMNAP was separated by Sephadex G-50 superfine via centrifugation (1000 rpm for 5 min at 4 °C). Moreover, to conjugate transferrin on liposomes surface, transferrin (TF) was firstly conjugated to Traut reagent (2-iminothiolane), (1:10 molar ratio) in HBS buffer (pH 8). Then, the reaction product was purified by Sephadex G-50 superfine hydrated with HBS pH 6.5. Finally, the Traut-TF conjugate obtained was then reacted overnight with the liposomal preparation. TF-liposomes formulation was then purified using Sephadex G-50 superfine hydrated in water.

Separately, calcium-phosphate colloidal dispersion (CaP NPs) was prepared by mixing dibasic hydrogen phosphate and calcium chloride solutions, followed by filtration. CaP NPs were then mixed to liposomes (1:1 v/v), obtaining SANP-TEMNAP and SANP-TF-TEMNAP. All formulation were prepared in triplicate (n = 3). Blank liposomes were also prepared and used as control.

### Physical-chemical characterization of SANP and SANP-TF encapsulating TEMNAP

The size, the polydispersity index and the zeta potential (ζ) were assessed by Zetasizer Nano Ultra (Malvern, UK). Results were derived from measurements conducted at minimum of three different batches (n = 3). To determine the amount of encapsulated TEMNAP, liposomes were diluted with acetonitrile (1:20) and TEMNAP was quantified spectrophotometrically at the λ of 231 nm. Results were expressed as encapsulation efficiency (EE%) (Figure 1C).

### BV2 microglial cell cultures

The immortalized murine BV2 cell line (ICLC ATL03001 Interlab Cell Line Collection, Banca Biologica e Cell Factory, Italy) was cultured in Dulbecco's Modified Eagle's Medium (DMEM, Invitrogen, Italy) supplemented with 10% fetal bovine serum (FBS), 1% penicillin-streptomycin (Invitrogen, Italy), and 1% glutamine (Invitrogen, Italy). Cultures were grown at 37 °C in 5% CO_2_ until 80% confluence.

### *In vitro* inflammatory stimuli and TSPO ligands treatments

The pro-inflammatory microglia state was induced by adding 100 ng/ml of LPS (Sigma-Aldrich) to the culture systems. The stimulations were performed in serum-free DMEM and experiments were generally stopped 24 h after the last stimulation. BV2 cells were treated with a final concentration of 100 ng/mL of LPS with or without of the new synthesized TSPO ligand TEMNAP at the concentrations of 10 nM, 50 nM or 100 nM. Cells were exposed to TEMNAP (100 nM) in free form, encapsulated in SANP (SANP-TEMNAP) or in SANP-TF (SANP-TF-TEMNAP). Control cells were exposed to vehicle or the PK11195 (Cat#5059810001, Merck Millipore) (100 nM), a well characterized TSPO ligand (39). BV2 microglial cells were pre-treated with the new synthesized TSPO ligand TEMNAP in free form or encapsulated and, 6 h after the pre-stimulation, immortalized microglia cells were co-incubated with the LPS for 24 h.

### Cell viability assay

Cell viability was assessed by biochemical CCK-8 kit for WST-8 assay (Dojindo, Kumamoto, Japan). BV2 cells (5x10^4^ cells in 100 µl/well) were seeded in 96-well plates. CCK8 solution (10 µl) was added to each well and the cultures were incubated at 37 ˚C for 90 min. The absorbance was measured at 450 nm and the cell viability was expressed as the percentage of control condition.

### Nitrite detection

Nitrite oxide (NO) generation in BV2 cells was determined using the Griess reaction assay as previously described 24, 25

### siRNA mediated TSPO gene silencing* in vitro*

Two different RNAi molecules targeting TSPO gene were tested, the Ambion™ Silencer™ Pre-Designed TSPO siRNA (Ref: AM16708, Thermofisher Scientific, USA) and the mm.Ri.Tspo.13.1 siRNA (IDT, Coralville, Iowa; USA). As negative control (siControl), the DS scrambled negative (IDT, Coralville, Iowa; USA) was used (Figure S2A).

BV2 cells were seeded in 60 mm culture dishes (400,000 cells/plate) and 35 mm culture dishes (40,000 cells/plate) and the two siRNAs were tested to a final concentration of 100 nM. As transfectant, RNAiMAX (Cat#13778-075, Thermo Fisher Scientific, USA) was used, according to manufacturer protocol.

The efficacy of siRNA strategies was evaluated at different timepoints (24, 48 and 72 h) after transfection and, the percentage of TSPO protein expression was calculated on biological triplicate by immunoblot followed by densitometric analysis.

Considering the significant reduction of TSPO protein expression (around 60%) observed when BV2 cells were exposed to TSPO siRNA (100 nM) for 72 h, these silencing conditions were chosen for all experiments in which TSPO silencing was necessary (Figure 6A).

After silencing TSPO protein expression in BV2 cell cultures with the selected siRNA strategy, the microglial cells were pre-treated with the SANP empty or with the nanoencapsulated-TEMNAP (SANP-TEMNAP) (100 nM) and, 6 h after TEMNAP pre-stimulation, immortalized microglia cells were co-incubated with the LPS for 24 h.

### Western blotting

Transfected BV2 cells were harvested and centrifuged. The RIPA Lysis Buffer (25 mM Tris-HCl, pH 7.6, 150 mM NaCl, 1% NP-40, 1% sodium deoxycholate, 0.1% SDS) supplemented with Halt™ Protease and Phosphatase Inhibitor Single-Use Cocktail (100X) (Thermo Fisher Scientific, USA) was added to each cell pellet to a final ratio of 2:1 v/v. After 15 min on ice, the suspensions were centrifuged (15,000 rpm) at 4°C for 30 min and the supernatants were collected in new tubes.

Bradford assay was used to quantify protein by Bio-Rad Protein Assay Dye Reagent Concentrate (Cat#5000006, Bio-Rad Laboratories, USA) and subsequently, each sample (30μg) supplemented with 2x Laemmli Sample Buffer (Cat#1610737, Bio-Rad Laboratories, USA) were boiled at 100°C for 5 min. Finally, the samples were loaded on a 12% precast 12% Mini-PROTEAN TGX Stain-Free Gel (Cat#4568044) and transferred on a nitrocellulose membrane using the Trans-Blot Turbo Transfer System (Cat# 690BR024275, Bio-Rad Laboratories, USA).

Membranes, blocked with 5% milk in TBS-T containing 0.1 % Tween-20 for 1 h, were incubated overnight at 4°C with anti-Vinculin (Sigma-Aldrich, Cat#V9264, RRID: AB10603627) and with anti-PBR (EPR5384) (Abcam, Cat#ab109497, RRID: AB 10862345). After secondary antibodies incubation, protein imaging was performed using an automated ChemiDoc™ MP Imaging System (Cat#12003154, Bio-Rad Laboratories, USA) and Clarity Max™ Western ECL Substrate (Cat# 1705062, Bio-Rad Laboratories, USA). Densitometric analysis was performed using ImageJ to quantify band intensities.

### Cytokines antibody array

Cytokine expression in cell culture media was evaluated by using the mouse cytokine antibody array membranes (Abcam, ab133994). Briefly, the media of LPS-stimulated BV2 cells treated with the SANP-TEMNAP (100 nM) in presence of siTSPO (100 nM) or siControl (100 nM) were collected. The cytokine array membranes were blocked by incubating with the blocking buffer at room temperature for 30 min. After the overnight incubation with the collected culture media, the membranes were washed by using the wash buffer I, the wash buffer II and incubated with the biotin-conjugated anti-cytokines at room temperature for 3 h. Then, the membranes were washed and incubated with HRP-conjugated streptavidin at room temperature for 3 h. The array membranes, transferred onto a plastic sheet, were incubated with the detection buffers mixture at room temperature for 2 min. Then, the membranes, sandwiched between two plastic sheets, were transferred to the imaging system and exposed. Imaging was performed using an automated ChemiDoc™ MP Imaging System (Cat# 12003154, Bio-Rad Laboratories, USA) and densitometric analysis was performed using ImageJ to quantify spot intensities.

### Animals

C57BL/6 mice and male Swiss Jim Lambert (SJL) mice were purchased from Charles River (Calco, Italy). Transgenic Tg2576 mice expressing the Swedish mutation (K670N and M671L), a human mutant form of the amyloid precursor protein (APP) (25), were purchased from Taconic (Germantown, NY,USA). Animals were housed in a temperature- and humidity-controlled room under diurnal/lighting conditions. All animal experiments and animal handling and care were in accordance with the ARRIVE guidelines and the Guide for the Care and Use of Laboratory Animals. Experiments were performed according to international guidelines for animal research, and the experimental protocol was approved by the Animal Care Committee of the "Federico II" University of Naples.

For immunohistochemical analysis, mice aged 9 months were deeply anesthetized and transcardially perfused with 4% (wt/vol) paraformaldehyde in phosphate buffer as previously described 26, 27. For immunohistochemical analyses, as well as for behavioural studies, mice were divided into four experimental groups: (i) SANP-TF wild-type mice; (ii) SANP-TF Tg2576 mice; (iii) SANP-TF-TEMNAP wild-type mice; and, (iv) SANP-TF-TEMNAP Tg2576 mice.

For *ex vivo* glia cultures, mice were euthanized by sevoflurane overdose, brains were quickly removed and dissected as below detailed. For immunocytochemical *ex vivo* studies mice were divided in two experimental groups: (i) SANP-TF Tg2576 mice; and (ii) SANP-TF-TEMNAP Tg2576 mice. The groups 'SANP-TF' received the nanoparticles empty, while the groups 'SANP-TF-TEMNAP' received the nanoparticles containing the new test compound TEMNAP.

### Genotyping of mice

Genomic DNA isolation from mouse tails and genotyping were performed as previously described 24. In brief, tails were cut and incubated in a digestion buffer (50 mM Tris-HCl pH 8.0, 100 mM EDTA pH 8.0, 100 mM NaCl, and 1% SDS) supplemented with 0.5 mg/mL Proteinase K (Sigma, Milan, Italy); next, they were placed in a shaking water bath at 55°C-60°C overnight. The polymerase chain reaction primers used to amplify the DNA region of the human APP Swedish mutation were 5′-CTGACCACTCGACCAGGTTCTGGGT-3′ and 5′-GTGGATAACCCCTCCCCCAGCCTAGACCA-3′ (Eurofins Genomics, Ebersberg, Germany) (Primm, Milan, Italy). The DNA (50 ng/μL) was then used for polymerase chain reaction. The amplification protocol (30 cycles) was the following: 95 °C for 45 s, 55°C for 60 s, and 72°C for 60 s. Each 25 μL reaction contained 1U of AmpliTaq DNA Polymerase (Lucigen, USA) and 0.5 μM of each primer. The amplification products were visualized on a 2% agarose gel electrophoresis by loading approximately half of each reaction per lane. The presence of the 466 bp indicated the transgenic genotype.

### Intraperitoneally administration of transferrin-conjugated nanoencapsulated TEMNAP (SANP-TF-TEMNAP)

SANP-TF and SANP-TF-TEMNAP, obtained as previously described, were intraperitoneally (i.p.) administered (20 mg/ml, 100 ml, daily for 4 weeks). The experimental groups were randomized as following: (i) Wild-type mice chronically treated with SANP-TF (Group 1); (ii) Wild-type mice chronically treated with SANP-TF-TEMNAP (Group 2); (iii) Tg2576 mice chronically treated with SANP-TF (Group 3); (iv) Tg2576 mice chronically treated with SANP-TF-TEMNAP (Group 4). Mice were subjected to daily administration of the respective formulations for four weeks starting seven months after birth, and they were examined 30 days after the end of i.p. administration. In vivo dose has been chosen on the basis of in vitro studies using in vitro in vivo correlations (IVIVC). Mice were subjected to daily administration of the chosen formulation for four weeks starting seven months after birth, and they were examined 30 days after the end of i.p. administration. The in vivo dose of 8 mg/100 g in a volume of 400 ml/100 g of mice body weight was chosen considering that in our in vitro experiments the highly effective concentration was 100 nM of TEMNAP (MW 450).

### Mass spectrometry detection of TEMNAP, after intraperitoneally administration of SANP-TF-TEMNAP

Frozen tissue samples harvested 24 h after i.p. treatment were pooled and weighed prior to extraction. The extraction procedure was adapted from the method described by Abujrais *et al.* 28 with minor modifications. Briefly, protein precipitation was achieved by adding cold methanol (15% DMSO) and 0.1% formic acid to the samples. Following homogenization, samples were incubated at -20°C for 30 min. The samples were then centrifuged, and the supernatant was collected. The supernatant was evaporated to dryness under a nitrogen stream and subsequently reconstituted in 100 μL of methanol (15% DMSO). To further purify the extracts, samples were centrifuged, and the supernatant was transferred to fresh tubes for storage at -20°C. Prior to analysis, 50 μL of each sample was mixed with 50 μL of a 50:50 (v/v) water solution containing 0.1% formic acid for injection.

The LC-MS/MS platform consisted of a UHPLC (Nexera Series LC-40, Shimadzu, Kyoto, Japan) coupled to a triple quadrupole/linear ion trap tandem mass spectrometer (QTRAP 4500, AB Sciex, Framingham, MA, USA) equipped with a Turbo V ion source. Instrument control, data acquisition, and processing were performed using the associated Analyst 1.6 software. The LC separation was carried out on a C18 column Gemini (2 mm x 50 mm, particle size 3u) from Phenomenex (Torrance, CA, USA). Elution was performed at a flow rate of 0.5 mL/min with water containing 0.1% (v/v) formic acid as eluent A and ACN (Merck) containing 0.1% (v/v) formic acid as eluent B, employing a linear gradient from 30% to 100% B in 7 min, and hold the solvent concentration for 6 min, and then back to initial condition in 2 min. The injection duty cycle was 20 min, including the column equilibration time. Q1 resolution was adjusted to 0.7 amu fwhm for MRM, referred to as the unit resolution. Q3 was also set to the unit resolution in MRM mode. MS analysis was carried out in positive ionization mode using an ion spray voltage of 5500 V. The nebulizer and the curtain gas flows were set at 35 psi using nitrogen. The Turbo V ion source was operated at 400°C with the Gas 1 and 2 flow (nitrogen) set at 50 psi. Two suitable MRM transitions were selected for the TEMNAP, and a Scheduled MRM method was developed. The compound-dependent parameters were optimised using the manual optimisation protocol in tuning mode. The Declustering Potential (DP) was set to 70, Exit Potential (EP) was set to 8.5, while the Q1 mass, the Q3 transition, and the best parameters are reported in Table 1.

A calibration curve was obtained, reporting the response area against the injected nanograms of TEMNAP. Ten microliters of each standard solution were injected in triplicate to build the calibration curves, and the concentration range used was from 0.04 to 8 ng.

### *Ex vivo* microglial cultures

Primary *ex vivo* microglia cultures were isolated from hippocampal regions of Tg2576 mice chronically treated with SANP-TF or with SANP-TF-TEMNAP. The hippocampal tissues were first digested enzymatically in a solution containing 0.125% trypsin and 1.5 mg/mL DNAse (Sigma, Milan, Italy) and then, the tissues were mechanically dissociated. Cell pellets were resuspended and plated onto poly-D-lysine-coated coverslips and primary microglia cultures were maintained in glia culture medium, as previously described 27.

### Confocal immunofluorescence analysis

Confocal immunofluorescence procedures in cells or sections were performed as previously described 26, 27. Brains were cryoprotected in sucrose, frozen, and sectioned coronally at 50 μm on a cryostat. Cell cultures were fixed in 4% wt/vol. paraformaldehyde in phosphate buffer for 30 min. After blocking with BSA 3%, cells or sections were incubated with primary antibodies for 24 or 48 h, respectively. The primary antibodies used were the following: mouse monoclonal anti-Iba1 (NCNP24) (Wako, Cat#016-26721, RRID:AB_2811160); rabbit anti-Iba1 (Wako, Cat#019-19741, RRID:AB_839504); rabbit anti-iNOS (EPR16635) (Abcam, Cambridge, UK, Cat#ab178945, RRID:AB_2861417); monoclonal mouse anti-iNOS (NOS-IN) (Sigma-Aldrich, St. Louis, Missouri, USA, Cat#N9657, RRID:AB_260818); mouse monoclonal anti-CD86 (B7-2 (D-6)) (Santa Cruz Biotechnology, Dallas, Texas, USA, Cat#sc-28347, RRID:AB_627200), rabbit polyclonal CD11b (Novus, Italy, Cat#NB110-89474, RRID:AB_1216361), mouse monoclonal IL1-Ra (A4) (Santa Cruz Biotechnology, Dallas, Texas, USA, Cat#sc-374084, RRID: AB_10917936). Then, cells or sections were incubated with fluorescence-labeled secondary antibodies. For double immunofluorescence, cells or sections were incubated in a mixture of fluorescent-labeled secondary antibodies (Alexa 488- and Alexa 594-conjugated anti-mouse or anti-rabbit immunoglobulins G). The fluorescent DNA-binding dye Hoechst-33258 was used to stain nuclei. Images were observed using a Zeiss LSM 700 laser (Carl Zeiss, Germany) scanning confocal microscope. Digital images were taken with an optical thickness of 0.7 μm and a resolution of 1,024 × 1,024 with 20× 40× or 63× objectives.

### Morphological and quantitative confocal studies

Morphological and quantitative studies *in vitro* were performed in single and double-labeling experiments. Iba1-positive microglia morphology was evaluated in single immunolabeled experiments and only cells with clearly a visible cell body were counted. Iba1-positive microglia were scored according to their morphological complexity in three categories: (i) resting, including cell with a flat and ramified morphologies; (ii) primed, including cells with few (one or two) branches or (iii) hyperactivated, including cell characterized by amoeboid and round shapes. Cell counting analyses of cell nuclei stained by Hoechst and quantification of Iba1^(+)^ positive microglia were performed manually. Only cells with detectable immunoreactivities within the cell body were counted.

Quantitative immunohistochemical studies *in vivo* were performed on digital images obtained from double-labeling experiments on collected images within the hippocampus of Tg2576 mice treated with SANP-TF or with SANP-TF-TEMNAP. For *in vitro* and *in vivo* quantitative analysis, digital images were taken with 40× and 63× objectives. The fluorescence intensities of Iba1, CD86 and iNOS as well as the percentage of iNOS-positive area were quantified respectively in pixel intensity and in percentage of immunostained area by the NIH image software 28-34. In order to perform quantitative confocal studies, identical exposure times and laser power settings were applied to all the photographs from each experimental set.

### Sholl analysis and microglial arbor morphometry *in vivo*

Sholl analysis was performed by using the ImageJ plugin Sholl Analysis 29, 30. Confocal images of immunohistochemical staining against Iba1 were used for tracing individual microglial soma and arbor of selected cells in ImageJ (Fiji) software 30. After assigning the center of each cell soma, a grid with concentric circles with increasing diameter (1 µm) was superimposed, as previously described 27. Next, numbers of intersecting points between concentric circles were counted to evaluate the complexity of cells. Data are expressed as the mean ± SEM of the values obtained in 3 independent experiments; SANP-TF n = 18 SANP-TF-TEMNAP n = 17.

The binary images of individual Iba1-positive microglial cells were obtained by using ImageJ (Fiji) software 31*.* The quantitative evaluation of cell morphology was performed by analyzing several parameters: (i) ending radius, i.e. the radius of the largest circle of the superimposed Sholl mask; (ii) intersecting radii, i.e., the number of radii of the superimposed Sholl mask intersecting the cells; (iii) sum of intersections, i.e., the sum of all intersections between the microglial arbors and the concentric circles radiating from the cell body; (iv) primary branches, i.e., processes emerging directly from the soma per cell; (v) branching point, i.e., the point at the processes where a process ramifies into two or more; (vi) soma area (in µm^2^), i.e., cross sectional surface area of the cell body; (vii) microglial arbor field area (in µm^2^), i.e., the number of pixels enclosed by the convex polygonal that is formed by connecting the tips of the longest processes 30. The numbers of primary branches, as well as the numbers of processes and branching points, were counted manually. ImageJ Fiji software was used to measure soma size and microglial arbors field area. The sum of intersections and the ending radius were measured using the Sholl method.

### Behavioral studies

Wild-type and Tg2576 mice were analyzed for cognitive behavioral performance at different time points after the chronic treatment with SANP-TF or with SANP-TF-TEMNAP. Behavioral tests aiming to evaluate memory and learning were performed at 9 months of age. Experiments were assessed under diurnal lighting conditions between 2:00 p.m. and 5:00 p.m. The memory and learning evaluations were performed with the following behavioral tests: (i) T-Maze tests; (ii) Novel Object Recognition and (iii) Barnes Maze tests.

***T-Maze spontaneous alternation.*** The T-Maze test was carried out in an enclosed "T"-shaped apparatus in which long arm of the T (47cm×10cm) serves as a start arm and the short arms of the T (35cm×10cm) serve as the goal arms. In this task the mouse was placed in the start arm and after 5 s the door was opened, and the mouse was allowed to choose and explore one of the goal arms. When the mouse had fully entered in the chosen arm (tail tip all the way in) a guillotine door was closed, and the mouse was confined to the chosen arm for 30 s. The mouse was then removed, the guillotine door was lifted, and the next trial initiated. This was repeated for a total of 10 trials per each mouse. At the conclusion of each trial the maze was cleaned of urine and feces 32, 33.

***Delay-dependent one-trial object recognition task***. The behavioral tests used in this study for assessing cognitive function were carried out in arenas (50 × 50 × 40 cm) resting on an infra-red emitting base. Mice behavior was recorded by an infrared-sensitive camera placed 2.5 m above the arenas and the data were analyzed using the tracking program Noldus. The object recognition task is based on the propensity of mice to explore a novel object versus a previously experienced object (familiar) when allowed to explore freely. Briefly, two identical objects were placed in the arena and animals were allowed to explore them for 10 min. Testing occurred 24 h later in the same arena in which one of the original objects was replaced by a novel object for 5 min. Object exploration was assumed as time spent in approaching an object (touching it with either mouse vibrissae, snout, or forepaws). The percentage of time spent exploring the novel object, compared with the total time spent exploring both objects, was considered as a measure of object recognition: discrimination index = t novel/(t novel + t familiar) X 100.The values are expressed as percentage of the obtained results. Videotracking of animals was analyzed by using EthoVision XT16 software (Noldus) 32.

***Barnes circular maze task.*** The Barnes test was performed by using a white circular platform (1.22 m diameter) elevated 40 cm above the floor and characterized by 32 equally spaced holes (5 cm diameter) around the periphery (5 cm from the platform perimeter). As previously described 34, only one hole led to a dark escape box (5 cm×5 cm×11 cm). The dark box was fixed in relation to the distal environmental cues and contained some dust-free sawdust bedding which was changed between trials. Mice were gently picked up from the tail and placed in the middle of the platform. The direction they faced at the start position was random and changed from trial to trial. After 5 min, if the mice did not find the goal box, they were gently directed toward the correct hole and allowed to descend into the escape box where they were left for 1 min. The following five test trials (one trial per day) were performed under the same conditions. Each trial ended when the mouse entered the goal escape box or after a maximum time of 5 min. The amount of time taken to enter the escape hole (escape latency) was noted 34.

### Statistical analysis

Sample sizes were chosen based on previous experience and considering similar studies from scientific literature. To ensure reproducibility, at least three independent replicates (n) for each experimental set were conducted *in vitro*. Number of independent experimental replicates (n) are indicated in figure legends. Sample processing and quantification in microscopy studies were performed in a blinded manner. For behavioral and confocal *in vivo* studies, considering a level of significance of p < 0.05 we used 5-8 mice/group determined by G-power software. The animals were assigned to different groups by random selection and no animals were excluded from the analysis. The data are expressed as the mean ± SEM of the values obtained from individual experiments. Statistical comparisons between groups were performed by Student's t-test or one-way analysis of variance (ANOVA) followed by Newman-Keuls or Bonferroni *post hoc* test. Differences of p < 0.05, p < 0.01 and p < 0.001 were considered significant. GraphPad Prism 6.0 was used for statistical analysis (GraphPad Software, Inc, La Jolla, CA, USA).

## Supplementary Material

Supplementary figures.

## Figures and Tables

**Table 1:** Optimized Q1 mass, transitions and parameters for MRM experiment. aCollision Energy. bCollision Exit Potential.cRetention Time

Metabolite	Precursor ion (*m/z*)	Product ion (*m/z*)	CE^a^	CXP^b^	RT^c^ (min)
TEMNAP	514.8 (M +H)^+^	285.0	18	15 11	8.15
		302.6	18	15 11	8.15

## Abbreviations

AD: Alzheimer's disease
APP: amyloid precursor protein
BBB: blood-brain barrier
CaP NPs: calcium-phosphate colloidal dispersion
CE: collision energy
CNS: central nervous system
CXP: collision exit potential
DMEM: Dulbecco's modified eagle's medium
DP: declustering potential
EE: encapsulation efficiency
EP: exit potential
FBS: fetal bovine serum
i.p.: intraperitoneally
iNOS: inducible nitric oxide synthase
IVIVC: *in vitro in vivo* correlations
LPS: lipopolysaccharide
MS: mass spectroscopy
NO: nitric oxide
NOS: nitric oxide synthase
NFKB: nuclear factor-kappa B
NSAID: nonsteroidal anti-inflammatory drug
PET: positron emission tomography
RT: retention time
SANP: self-assembling nanoparticle
SANP-TF: transferrin-conjugated self-assembling nanoparticles
TEMNAP: 7-chloro-1-methyl-2-oxo-5-phenyl-2,3-dihydro-1H-benzo(e)(1,4)diazepin-3-yl2-(6-methoxynaphthalen-2-yl) propanoate
TF: transferrin
TfR: transferrin receptors
TSPO: mitochondrial translocator protein 18kDa
ζ: zeta potential
