# Supplementary material for: A new nano-encapsulated TSPO ligand reduces neuroinflammation and improves cognitive functions in Alzheimer's disease model
Source: Theranostics. 2025 Mar 21;15(10):4673–92. doi: 10.7150/thno.106083 (PMC11984405; doi:10.7150/thno.106083)

SUPPLEMENTAL MATERIALS

Figure S1. Morphological assessment of Iba1-positive BV2 microglia after exposure to TEMNAP [100nM]

(A) (a-c) Confocal microscopic images displaying three representative morphological categories of Iba1$^{(+)}$-positive cells: resting (a), primed (b) and hyperactivated (c). Nuclei were stained by the nuclear dye Hoechst-33258. Scale bars: 5µm.

(B) Quantitative morphological analysis of Iba1$^{(+)}$-positive microglia in control condition or exposed to LPS in presence respectively of: (i) self-assembling nanoparticle (SANP); (ii) nanoencapsulated-TEMNAP [100nM] (SANP-TEMNAP); (iii) TEMNAP [100nM] non-nanoencapsulated. Iba1$^{(+)}$-positive cells were scored in three categories accordingly to their morphological complexity: (i) resting; (ii) primed; and (iii) hyperactivated. In each category data were normalized on the total number of Iba1$^{(+)}$-positive cells. The values represent the mean±SEM; (n=3 indipendent experimental replicates); *p<0.05 versus respective LPS-SANP condition; #p<0.05 versus respective LPS-TEMNAP [100nM] condition.

Supp. Figure 1

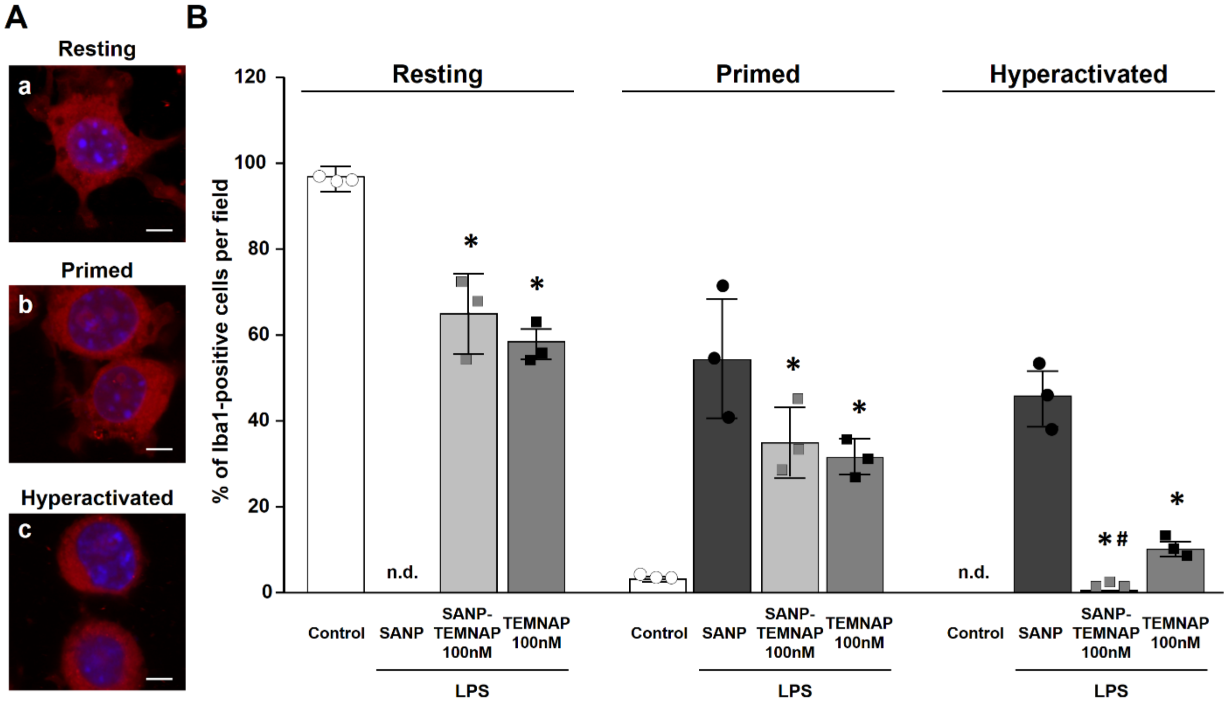

Figure S2. Kinetics of two selective siRNA targeting TSPO in BV2 microglial cells

(A-B) Images showing two representative blotting of TSPO protein expression levels in BV2 microglial cells exposed respectively to two different RNAi molecules targeting TSPO gene: **(A)** The Ambion™ Silencer™ Pre-Designed TSPO siRNA (#1) and **(B)** the mm.Ri.Tspo.13.1 siRNA (#2) at the concentration of [100nM] or to siControl [100nM]. BV2 microglial cells were harvested at the indicated time points (48h and 72h). The vinculin was used as housekeeping protein.

Supp. Figure 2

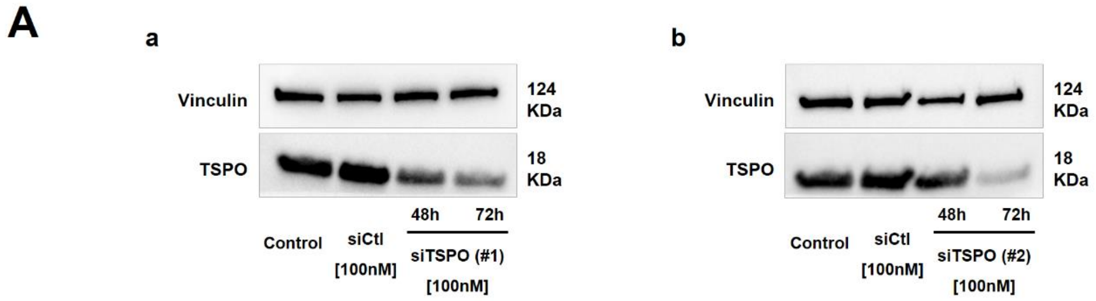

Figure S3. Example of Chromatograms obtained from brain cortex homogenates deriving form mice intraperitoneally infused with: (A) Negative Control; (B) Only Nanoparticles; (C) SANP-TF-TEMNAP.

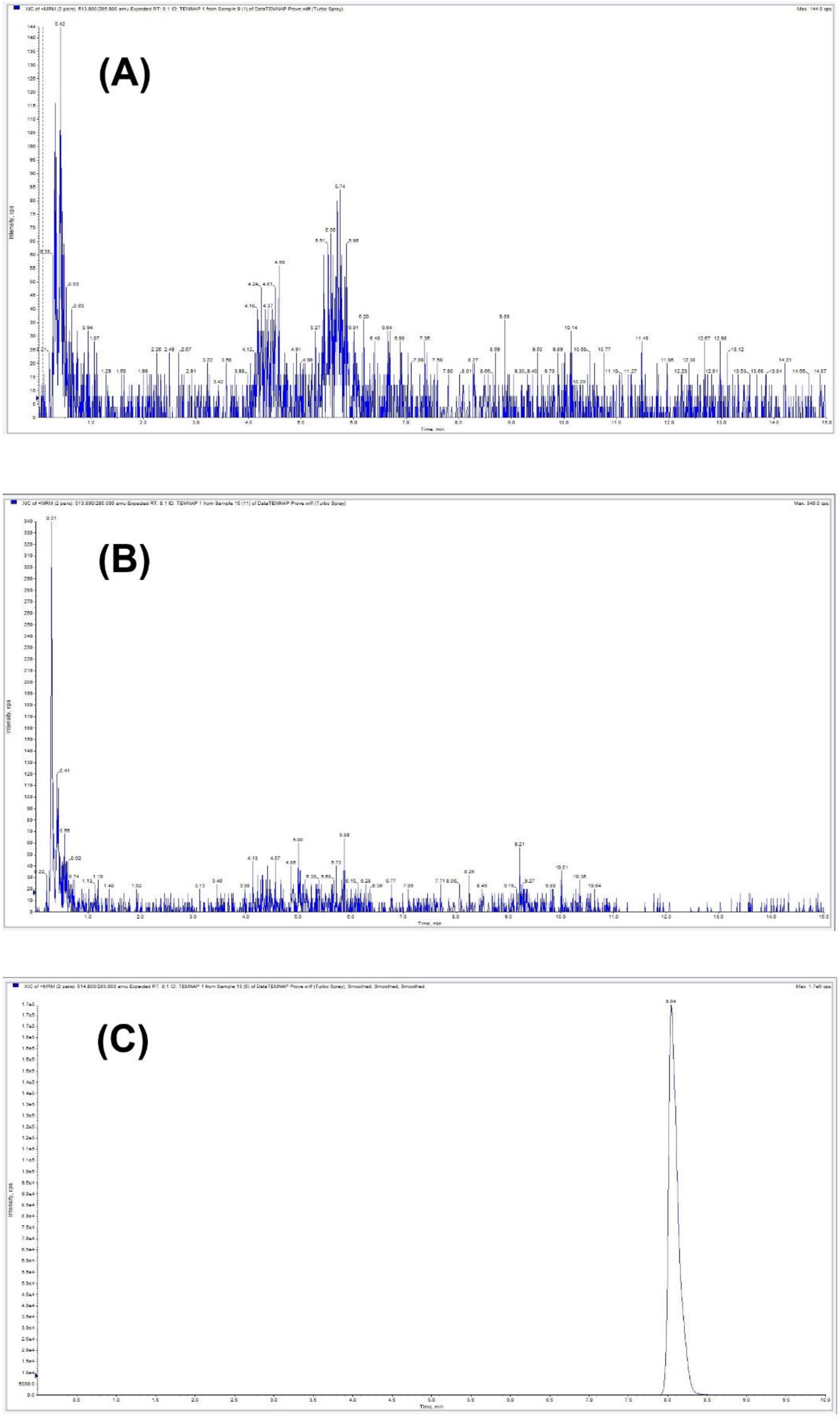

Supplement: Supplementary file 1 — Supplementary figures. [file thnov15p4673s1.pdf]
